# Coexisting Coronary and Carotid Artery Disease: What We Did, What Happened

**DOI:** 10.21470/1678-9741-2021-0127

**Published:** 2022

**Authors:** Mehmet Raşit Güney, Erhan Güler, Erkan Albay, Tamer Kehlibar, Mehmet Yilmaz, Bülend Ketenci

**Affiliations:** 1 Department of Cardiovascular Surgery, Dr Siyami Ersek Thoracic and Cardiovascular Surgery Training and Research Hospital, Istanbul, Turkey.; 2 Department of Cardiovascular Surgery, Cleveland Clinic Miller Family Heart and Vascular Institute, Cleveland, Ohio, United States of America.

**Keywords:** Coronary Artery Bypass, Carotid Endarterectomy, Carotid Arteries, Transient Ischemic Attack, Progression-Free Survival, Myocardial Infarctation, Stroke

## Abstract

**Introduction:**

There is no complete consensus on the three surgical methods and long-term consequences for coexisting coronary and carotid artery disease. We retrospectively evaluated the surgical results in this high-risk group in our clinic for a decade.

**Methods:**

Between 2005 and 2015, 196 patients were treated for combined carotid and coronary artery disease. A total of 50 patients were operated on with the staged method, 40 of which had carotid endarterectomy (CEA) priority, and 10 had coronary artery bypass grafting (CABG) priority. CABG and CEA were simultaneously performed in 82 patients; and in 64 asymptomatic patients with unilateral carotid artery lesions and stenosis over 70%, only CABG was done (64 patients). Results were evaluated by uni-/multivariate analyses for perioperative, early, and late postoperative data.

**Results:**

In the staged group, interval between the operations was 2.82±0.74 months. Perioperative and early postoperative (30 days) parameters did not differ between groups (P-value < 0.05). Postoperative follow-up time was averaged 94.9±38.3 months. Postoperative events were examined in three groups as (A) deaths (all cause), (B) cardiovascular events (non-fatal myocardial infarction, recurrent angina, congestive heart failure, palpitation), and (C) fatal neurological events (amaurosis fugax, transient ischemic attack, and stroke). When group C events were excluded, event-free actuarial survival rates were similar in all three methods (P=0.740). Actuarial survival rate was significantly different when all events were included (P=0.027). Neurological events increased markedly between months 34 and 66 (P=0.004).

**Conclusion:**

Perioperative and early postoperative event-free survival rates were similar in all three methods. By the beginning of the 34^th^ month, the only CABG group has been negatively separated due to neurological events. In the choice of methodology, “most threatened organ priority’’ was considered as clinical parameter.

**Table:** Abbreviations, Acronyms & Symbols

ANOVA	= Analysis of variance	EF	= Ejection fraction
CABG	= Coronary artery bypass grafting	LMC	= Left main coronary
CAS	= Carotid artery stenosis	MI	= Myocardial infarction
CEA	= Carotid endarterectomy	NYHA	= New York Heart Association
CI	= Confidence interval	PMI	= Perioperative myocardial infarction
COPD	= Chronic obstructive pulmonary disease	PVD	= Peripheral vascular disease
CPB	= Cardiopulmonary bypass	TIA	= Transient ischemic attack

## INTRODUCTION

In the presence of high genetic predisposition and risk factors, severe atherosclerotic disease can be seen in more than one system at the same time. Dual coronary and carotid artery involvement may increase from 8% to 18% in parallel with the number of risk factors in the patient^[1]^. Surgical approaches to this dual system involvement are various and are still open for discussion. It is reasonable to think that the revascularization of one system can have negative effects on another. Current data has shown that stroke risk can increase from 1.3% to 14% in combined disease compared with the isolated coronary artery bypass grafting (CABG)^[2,3]^. Similarly, the risk of perioperative myocardial infarction (PMI), which is 0.5-1.5% in isolated carotid endarterectomy (CEA), can increase up to 17-20% in in combined disease^[4,5]^. This reality has encouraged the search for a way to combine the two surgical procedures to minimize risks for the patient. The resultant combined interventions were classified as either simultaneous (the two surgical procedures done in the same session under a single anesthetic process) or staged with a short period of time between procedures (usually < 6 months), being the first procedure CABG (staged) or CEA (reverse staged). Some authors also argue that leaving untouched asymptomatic unilateral cases with severe stenosis (> 70%) may be safe^[6,7]^.

In this study, we retrospectively evaluated the simultaneous, staged, and only CABG methods employed in the management of dual coronary/carotid disease in a single center from 2005 to 2015 and their long-term follow-up results.

## METHODS

This study included 196 patients operated for dual carotid/coronary disease between 2005 and 2015. Patients with preexisting chronic atrial fibrillation, stroke patients, patients with hybrid interventions (carotid stenting plus CABG), and emergency operations were excluded. All CABG candidates underwent routine carotid artery screening (Doppler ultrasound and/or angiography). CABGs, in all patients, were performed using the standard protocols — following midline sternotomy, cardiopulmonary bypass (CPB) was established by utilizing standard aortic and two-staged venous cannula. Cardiac arrest was ensured through antegrade isothermic blood cardioplegia^[8,9]^, topical cooling, and moderate hypothermia (28 °C). In high-risk patients (n=110 [56%]) with extensive atherosclerosis, proximal anastomoses were accomplished by side-clamping if the ascending aorta was safe enough to permit it. Proximal anastomosis in the remaining patients was performed during a single cross-clamping. In the simultaneous method (n=82), head and neck regions were included to the sterilization process; CEA was carried out before CPB while harvesting of the left internal mammary artery. We used carotid shunt if stump back pressure was < 50 mmHg. We performed carotid revascularization with endarterectomy, graft interposition, or end-to-side bypass methods. In all conventional CEAs, we used the saphenous vein or prosthetic patch to provide a perfect reconstruction of the artery.

In the staged method (n=50), we gave priority to the organ perceived to have higher ischemic treat. If neurological symptoms dominated, we performed staged procedure with CEA first. When the cardiac symptoms were predominant (10 patients, 8 with left main coronary [LMC] disease), we performed reversed stage procedure with CABG first. The second operation was performed as soon as possible. In the staged group, CEA was made with local anesthesia in 17 patients (34%). In a previous study, we have shown that there were no significant differences in terms of surgical endpoints between general and local anesthesia groups in CEA patients^[10]^. The same surgical team performed all the operations.

In 64 patients with unilateral, serious (> 70%), but asymptomatic carotid artery stenosis, only CABG was performed. In all three groups, we did not allow cerebral perfusion pressure to drop < 60 mmHg, with vigorous intervention using volume replacement and bolus vasoconstrictors as required.

The major peri-postoperative events, transient ischemic attack (TIA), stroke, and PMI and postoperative myocardial infarction, were defined as follows.

We described TIA as a kind of stroke that lasted a few minutes, including numbness or weakness on one or both sides of the body. We accepted non-lateralizing deficits, cranial nerve involvement, dysarthria, and lacunar states as minor neurological sequelae with favorable prognosis if the Rankin score for the patient was ≤ 2. Motor hemiparesis/hemiplegia states, sensory motor stroke states, and hemispheric syndromes with a Rankin score ≥ 3 were all included in the definition of stroke with a worse prognosis.

PMI was considered to be present if electrocardiography revealed new Q wave > 0.04 s in two or more derivations or > 25% R loss, creatine phosphokinase-myocardial band > 100 IU/lt and troponin I peak 3.7 µg/lt or 3.1 µg/lt at the 12^th^ hour or 2.5 µg/lt at the 24^th^ hour.

In the postoperative period, patients were closely followed up for 10 days after surgery, and then checked at intervals in the first month, three months, and first year. We asked the patients to report any complaint immediately. The follow-up of patients residing in remote localities was provided by phone or local health institutions.

We obtained the requisite data for this study from the local hospital records and national databases like e-Nabız and Medulla systems. This retrospective study has been approved by the ethics committee of the Dr Siyami Ersek Thoracic and Cardiovascular Surgery Training and Research Hospital (47124).

The endpoint data used in the follow-up were (A) deaths (all causes), (B) cardiovascular events (non-fatal myocardial infarction, recurrent angina, congestive heart failure, palpitation [arrhythmia]), and (C) neurological events (amaurosis fugax, TIA, stroke).

### Statistics

All the accessed data were entered in the SPSS Inc. Released 2009, PASW Statistics for Windows, version 18, Chicago: SPSS Inc. software. The numerical data were reported as means ± standard deviations. Crosstab Pearson’s chi-squared test (non-numerical) and one-way analysis of variance (ANOVA) (numerical) analyses were utilized for calculations involving the three groups. In two-group comparisons, unpaired two-tailed Student’s *t*-test (numerical) was used; in non-numerical comparisons, two-by-two contingency tables were corrected according to Yates. When assumptions were violated for expected frequencies, Fisher’s exact test was used. Kaplan-Meier survival analysis was used for the event-free survival tables, and the differences in their distribution were evaluated by means of the log-rank test. The impact of the differences in preoperative risk factors in the groups on neurological events (stroke/TIA), which are differential endpoints, was calculated by utilizing multinominal regression analysis.

## RESULTS

**Fig. 1:**
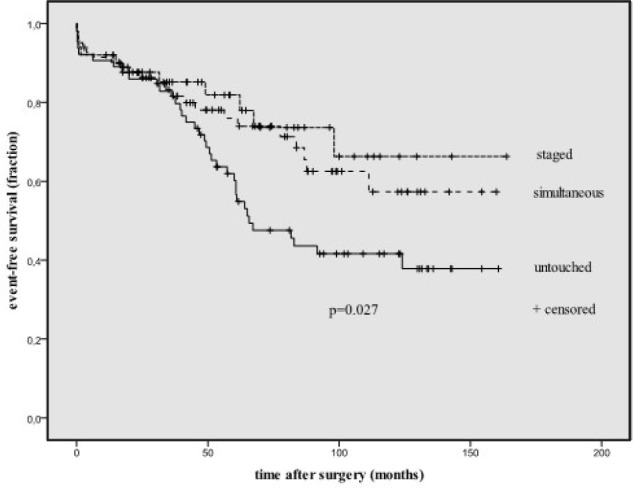
Event-free actuarial survival functions in the groups. Untouched=only coronary artery bypass grafting group

**Fig. 2:**
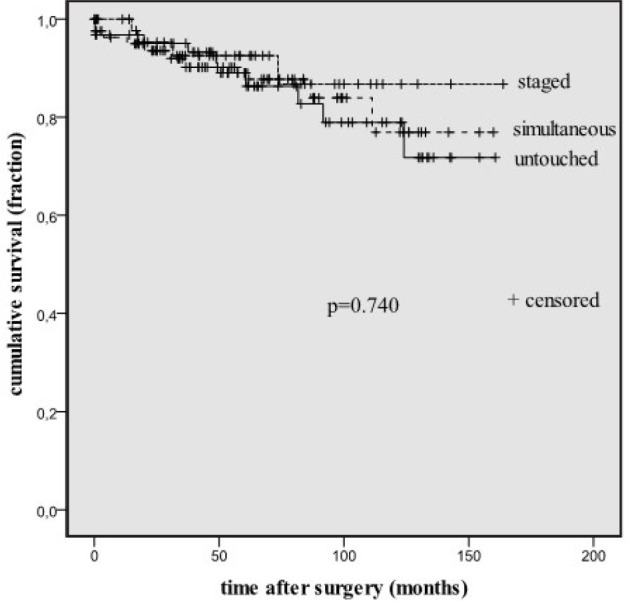
Cardiac-related event-free survival curves of groups. Untouched=only coronary artery bypass grafting

**Fig. 3:**
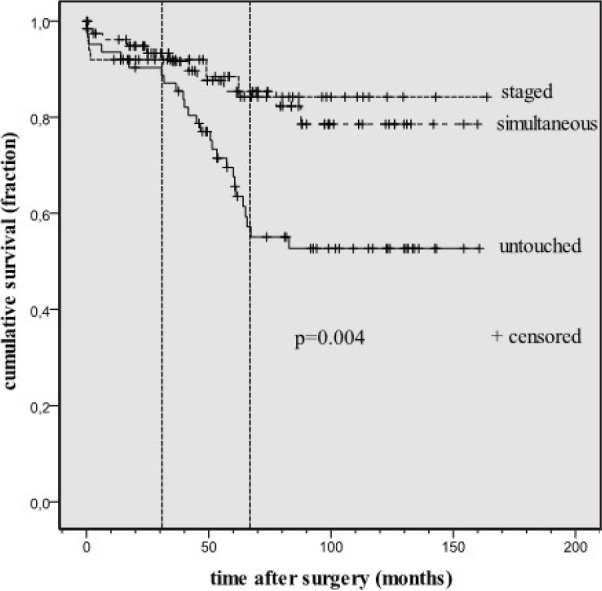
Neurological-related event-free survival curves of the groups. Untouched=only coronary artery bypass grafting

Preoperative demographic and clinical data are shown in Table 1. An important outcome in this table is that the LMC disease rate (23.5%) is relatively high compared to the incidence rate (3-9%) in normal population^[11,12]^. Smoking and LMC disease incidence in the simultaneous group were significantly higher with *P*-values 0.04 and 0.011, respectively. In the reversed staged group, eight of 10 patients (80%) had LMC disease. LMC disease patients in the simultaneous group also had neurological symptoms and/or near-total critical (90-99%) carotid stenosis (68 patients [83%]). The period between the two operations in the staged group was 2.82±0.74 months. CPB duration (*P*=0.503, one-way ANOVA), cross-clamping time (*P*=0.66, Pearson’s chi-squared), and mean number of distal anastomosis (*P*=0.646, one-way ANOVA) were not significantly different between groups. Early mortality and morbidity reported in Table 2 were not different between the three groups in the perioperative period (from operation to the first 30 days). Late follow-up duration was 94.9±38.3 months. The event-free actuarial survival curves of the groups for all-cause mortality and cardiovascular and neurological events are reported in Figure 1. Negative dissociation was observed in the only CABG group in terms of event-free survival (*P*=0.027). When neurological events were excluded, event-free survival differences between groups were lost (Figure 2, *P*=0.740). If cardiovascular events were excluded, the negative dissociation of the only CABG group re-emerged (Figure 3, *P*=0.004). Event-free survival deviation determined by vertical lines in Figure 3 begins in the postoperative 34th month (*P*-value > 0.05 at this point) and continues until the 66^th^ month postoperatively. In the only CABG group, 34 patients, whose degree of carotid stenosis and/or symptoms increased, underwent CEA in our hospital or in other local hospitals during the follow-up period. Two patients had stroke during this time and a few patients have still some atypical neurological symptoms.

**Table 1:** Patients’ demographical characteristics and preoperative cardiac and neurological clinical data.

Variables	Staged group (n=50)	Simultaneous group (n=82)	Only CABG group (n=64)	*P*-values
Age (years)	66.7±7.25	67.2±8.44	68.8±8.99	0.369^a^
Age > 70 years	18	31	30	0.418
Male	37	58	38	0.193
Hypertension	26	53	30	0.084
Smoking*	38 (76%)	53 (64%)	34 (53%)	0.041
Diabetes mellitus	10	27	18	0.276
Dyslipidemia	25	42	30	0.87
COPD	7	17	7	0.252
PVD	9	10	10	0.643
Renal dysfunction	1	3	4	0.507
Unstable angina*	15 (30%)	45 (55%)	27 (42%)	0.019
Prior MI	20	39	24	0.44
EF < 50%	20	37	28	0.845
NYHA III-IV	19	35	26	0.868
LMC disease*	8 (16%)	28 (34%)	10 (15%)	0.011
Three-vessel disease	39	67	53	0.796
Neurologically asymptomatic	26	51	N/A	0.278^b^
TIA	20	24	N/A	0.254^b^
Stroke	4	6	N/A	1.000^c^
Bilateral CAS	15	16	N/A	0.205^b^
Severe stenosis 90-99%	42 (84%)	59 (72%)	56 (88%)	0.120

>CABG=coronary artery bypass grafting; CAS=carotid artery stenosis; COPD=chronic obstructive pulmonary disease; EF=ejection fraction; LMC=left main coronary; MI=myocardial infarction; NYHA=New York Heart Association; PVD=peripheral vascular disease; TIA=transient ischemic attack

a aone-way analysis of variance;

b Pearson’s chi-squared test;

c Fisher’s exact test, remainings, crosstab ;

* *P*<0.05

**Table 2:** Operative in-hospital 30-day results.

Variables	Staged group (n=50)	Simultaneous group (n=82)	Only CABG group (n=64)	*P*-values
PMI	3 (6%)	3 (3.8%)	2 (3.1%)	0.720
TIA	0	1 (1.2%)	0	0.497
Stroke	2 (4%)	4 (4.87%)	1 (1.56%)	0.553
Death	3 (6%)	4 (4.87%)	2 (3.1%)	757
Death or stroke	5 (10%)	8 (9.75%)	3 (4.68%)	0.464

CABG=coronary artery bypass grafting; PMI=perioperative myocardial infarction; TIA=transient ischemic attack

None of the preoperative differences between surgical groups — LMC disease, *P*=0.720, Exp(B)=1.168, confidence interval (CI) bounds=0.501-2.722; unstable angina, *P*=0.728, Exp(B)=1.130, CI bounds=0.568-2.249; and smoking, *P*=0.832, Exp(B)=0.925, CI bounds= 0.452-1.895 — had an effect on neurological endpoints, tested by multinominal regression analysis.

## DISCUSSION

In extensive diseases that affect multiple organs, we may deviate from standard surgical procedures or can plan hybrid interventions. In type 1 dissection, arcus debranching plus endovascular aneurysm repair, CABG plus ascending aorta or axillary-bifemoral bypass, and CABG plus renal/superior mesenteric/carotid artery stenting are some of the examples. Combined coronary and carotid artery disease are one of the most frequently seen combinations. Severe carotid stenosis is accompanied by coronary artery disease in 40-50% of patients^[13,14]^. For surgical treatment of these coexisting diseases, three approaches that we have retrospectively evaluated were considered. However, there is no consensus reported in these guidelines regarding the indications of these interventions. The only criterion we observed in our series was which organ (heart *vs.* brain) was more threatened. Although this opinion depended on certain criteria, it was still subjective. For example, in our series, 88% of only CABG patients had critical and near total carotid stenosis. Was it sufficient for patients to be neurologically asymptomatic and unilateral for this intervention to be chosen? Though it may appear controversial, there are wide and reliable series in the literature that give supportive evidence^[14-17]^. A complete methodology based on universally acceptable criterion is still out of our grasp.

In evaluating an organ under ischemic threat due to a chronic process, we must study the extent of collateralization. Collateralization in the heart is clearly defined as vascularization of the region fed by one epicardial coronary artery by another through anastomotic channels^[18]^, and these connections are thought to be natively present^[19,20]^. To assess these collaterals which may be inadequate, Rentrop Classification with angiography^[21]^, calculation of the collateral flow index with intravascular ultrasound application^[22]^, or intracoronary electrocardiogram^[23]^ can be done.

On the other hand, in cases of carotid artery occlusion, proper circulation can be maintained by the extensive collaterals from Circle of Willis, ipsilateral vertebral artery, ipsilateral thyrocervical trunk or costocervical trunk, ascending cervical artery or deep cervical artery, occipital artery, and ipsilateral superior and inferior thyroid artery^[24]^, which may be why total common or internal carotid artery chronic occlusions can remain asymptomatic and do not require surgical intervention with no damage and no clinical findings^[25]^. Because of that, we have no means of quantitating the adequacy of the collateral circulation in cerebral circulation.

In addition, despite all the advances in imaging methods, it is not possible to know exactly what is happening in microcirculation and predict its clinical and pathologic significance. It is also not possible to know which collaterals will remain open from birth and which will regress. Similarly, it is not known how reliably the collateral will work at the time of arterial occlusion.

A concrete result in this retrospective study was that in the perioperative and early postoperative period, survival and quality of life did not change regardless to method. However, by the 34^th^ month, neurologically based mortality and morbidity increased in patients in the only CABG group (Figure 3), but it remained similar in patients in other groups (Figure 2). The only other study on this particular issue conducted by Gaudino M. et al.^[26]^ reported similar results for their 139 patients. They observed an increase in neurological events in only CABG patients with severe asymptomatic and unilateral carotid stenosis at the mid-term follow-up period. They reported that those patients required CEA after a mean postoperative period of 46.5±11.1 months.

### Limitations

The retrospective design and relatively small sample size are the limitations of this study.

## CONCLUSION

In conclusion, multi-center prospective studies and meta-analyses are needed to form criteria dependent collateralization levels in the choice of management strategy preferring one of the three methods over the others to treat coexisting coronary and carotid disease. There is no significant difference for the early postoperative event-free survival in the three methods used in this study. However, we should closely follow up and monitor the only CABG group postoperatively. If any neurological symptom arises or the degree of carotid artery stenosis increases, treatment should be prompt.
